# Advantages of Evaluating Mean Nuclear Volume as an Adjunct Parameter in Prostate Cancer

**DOI:** 10.1371/journal.pone.0102156

**Published:** 2014-07-09

**Authors:** Eduardo Leze, Clarice F. E. Maciel-Osorio, Carlos A. Mandarim-de-Lacerda

**Affiliations:** Laboratory of Morphometry, Metabolism and Cardiovascular Disease, Biomedical Center, State University of Rio de Janeiro, Rio de Janeiro, Brazil; Innsbruck Medical University, Austria

## Abstract

**Background:**

Efforts to improve the diagnosis, prognosis and surveillance of prostate cancer (PCa) are relevant. Gleason score (GSc) overestimation may subject individuals to unnecessary aggressive treatment. We aimed to use stereology in PCa evaluations and investigate whether mean nuclear volume (MNV) correlates with the Gleason primary pattern (Gpp) and to improve the subjective GSc to obtain an objective and reliable method without inter-observer dissension.

**Methods:**

We identified 74 radical prostatectomy specimens that were divided into six groups based on Gpp, from 3 to 5. Controls (C) were designed in paired non-tumor regions of the same specimens. MNV was estimated using the “point-sampled intercepts” method. Differences in MNV among the C groups and the Gpp groups were tested with the Kruskall-Wallis test and Dunn post-hoc test. Differences between each Gpp group and its control counterpart were tested with the Wilcoxon test. Correlations were evaluated with the Spearman rank correlation (R_[Spearman]_).

**Results:**

The correlations between prostate-specific antigen (PSA) and GSc (R_[Spearman]_ of 0.76) and between PSA and MNV (R_[Spearman]_ of 0.78) were moderately strong and highly significant, and the correlation between MNV and Gpp (R_[Spearman]_ of 0.53) was moderate and highly significant. MNV was significantly greater in cancerous regions than in paired-control regions. Limitations included sample size.

**Conclusions:**

Proper planning of a study, as well as the availability of equipment and software for morphological quantification, can provide incentive to quickly and accurately estimate MNV as an adjunct parameter in the assessment of PCa. Current data are in favor of the use of MNV associated with GSc and PSA in the assessment of PCa.

## Introduction

Prostate cancer (PCa), which is an important public health concern in Western countries and an emerging malignancy in developing nations, is the most frequently diagnosed cancer in American males and the second leading cause of cancer deaths in the United States [1]. Thus, efforts to improve the diagnosis, prognosis and surveillance of PCa are relevant.

It is notable that nearly 25 years after its adoption, the Gleason score (GSc) system remains a timely method to evaluate the correlation between histologic grade and prognosis in patients with PCa [2]. This system is based on the architecture of cancer cells, which are assigned to one of five histologic patterns of decreasing differentiation. Since 2005, it has been recommended that pathologists assign a GSc grade by adding together the most common and the highest Gleason patterns in a biopsy, as opposed to the original GSc method that added the most common and the second most common patterns [3]. These modifications made by the International Society of Urological Pathology (ISUP) in 2005 were an attempt to improve the correlation between biopsy and radical prostatectomy, but there is also a need for a change in reporting to more closely reflect tumor behaviour [4].

Studies have shown that, given the poor inter-observer reproducibility of the GSc, it is difficult to achieve mutual concordance [5]–[7]. In addition, almost 20% of radical prostatectomy cases demonstrate tertiary patterns, which means that a needle biopsy sample exhibits a higher tertiary Gleason pattern, resulting in apparent overgrading of the needle biopsy [8]. This lack of agreement is primarily important in patients with PCa as well as individuals who are candidates for or are under active surveillance [9].

Without doubt, progress in terms of the identification of cancer molecular markers has benefited PCa diagnosis, prognosis and treatment [10], [11]. However, the nuclear structure in the tumor remains a matter of interest. Alterations in nuclear size and shape, in the number and size of nucleoli and to chromatin can be related to the altered functional properties of cancer cells or tissue remodelling [12]. Consequently, stereological estimation of the mean nuclear volume (MNV), which is an unbiased estimate of three-dimensional variables obtained from a two-dimensional set of images, represents an objective and reproducible grading method for PCa [13].

The aim of the present study is to revisit the use of stereology in PCa evaluation and investigate whether MNV correlates with the Gleason primary pattern (Gpp) to replace or improve the subjective GSc such that it becomes a more objective and reliable method without inter-observer dissension.

## Material and Methods

This study follows the principles outlined in the Declaration of Helsinki and was approved by the local ethics committee for scientific research (Ethics Committee for Research with Human Subjects at the State University of Rio de Janeiro, CEP, Process Number 618329) that is associated with the National Committee for Ethics in Research (CONEP) and directly linked to the National Health Council of Brazil.

We submitted the research to *Platform Brazil*, the national and unified basis of records of research involving humans for any CEP/CONEP system (http://conselho.saude.gov.br/). The material was collected over the years and filed in an institutional collection. Thus, at the moment of the research we had no more contact with the patients or their families. We have discussed frankly with members of CEP the difficulty of retrieving signatures in an written informed consent by patients in the present case. The members of the CEP have considered this point raised by us. In their decision, they emphasized the need of a written informed consent of patients, but they understood the present case as exceptional and, therefore, the study was exempted from informed consent of patients.

This study was performed on specimens obtained from 74 patients who underwent radical prostatectomy as a first-choice therapy for localized and locally advanced PCa. The Gpp was established by only one trained pathologist (CM-O). Patients were divided into six groups based on Gpp, and the prostate-specific antigen (PSA) value of each sample was noted prior to surgery. Three groups represented the primary pattern grades (tumor regions, T) of Gpp 3 (G3, n = 20), Gpp 4 (G4, n = 28), and Gpp 5 (G5, n = 26). Controls were designed in paired non-tumor (NT) regions of the same specimens and were referred to as C3 (for NT regions of Gleason 3 specimens), C4 (for NT regions of Gleason 4 specimens), and C5 (for NT regions of Gleason 5 specimens).

The material was rapidly fixed for 48 h in freshly prepared formaldehyde (4% wt/vol in 0.1 M phosphate buffer, pH 7.2), embedded in paraffin, sectioned with a nominal thickness of 5 µm, and stained with haematoxylin and eosin. The sections were analyzed using an oil immersion plan achromatic objective (×100) with an Olympus BX51 microscope (Olympus America, Miami, USA) and an Infinity 1–5c digital camera (Lumenera, Ottawa, ON, Canada). Digital images were taken for quantification of T and NT regions as defined by the expert pathologist.

### Stereology

**Figure 1:**
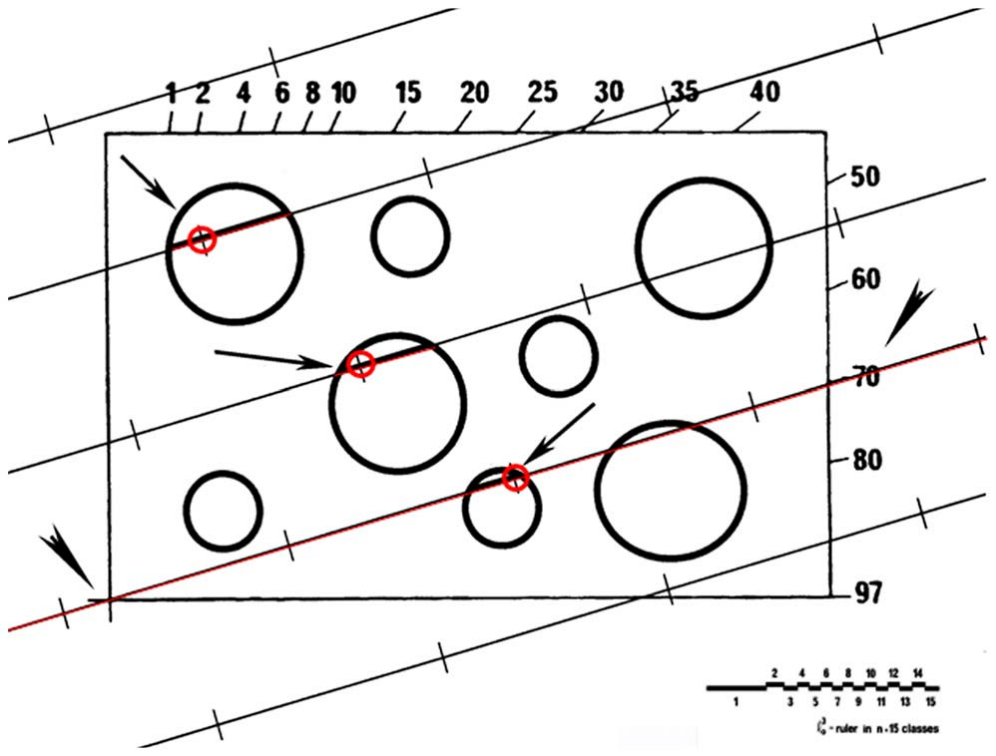
Schema of the test system used to estimate the mean nuclear volume based on the “point-sampled intercepts” method. The test system, with points, is overlaid on the prostate image and aligned with the border numbers as defined by lottery (arrowheads). The length of the sampled nuclei (arrows) is measured with a logarithmic ruler (see below).

MNV was estimated using the “point-sampled intercepts” method in five different microscopic fields per patient totalling at least 50 nuclei per specimen [14]. A test system consisting of parallel lines associated with test points was superposed onto each microscopic field as demonstrated in Figure 1. The direction of the lines on the sample was determined by lottery, and for each point inside the unbiased counting frame that hit a nucleus, the nuclear intercept through the point was measured. The measurement of the intercept length was performed using a 32-mm logarithmic ruler composed of a series of 15 classes, where the width of any class is approximately 17% larger than that of the preceding class. Each individual intercept was cubed, and the mean of all of these values was multiplied by π/3 to give MNV. The numerical nuclear density in the plane, i.e., the number of nuclear profiles per area (N_A_), was determined using a frame of 5,675 µm^2^.

### Statistical analysis

The Spearman rank correlation (R_[Spearman]_) was estimated for each sample to determine the correlations between: a) GSc and PSA, b) MNV and PSA, and c) Gpp and MNV (Tables S1 and S2). The differences in MNV between the groups and within the C and G groups were assessed by the Kruskall-Wallis analysis of variance followed by the Dunn post-hoc test. The differences that resulted when comparing each Gpp with its control counterpart were assessed by the Wilcoxon test. Data are shown as the median and the 95% confidence interval (CI). A *P-value* of ≤0.05 was considered statistically significant. The GraphPad Prism program (version 6.03 for Windows, GraphPad Software, La Jolla, CA, USA) was used to perform statistical analyses and generate graphics.

## Results

### Spearman rank correlations

**Figure 2:**
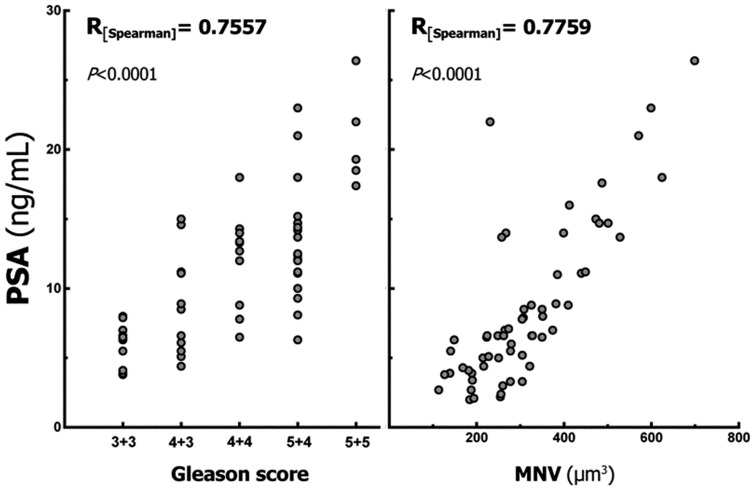
Correlations between the prostate specific antigen (PSA) value and both the Gleason score and mean nuclear volume (MNV). The Spearman coefficients of correlation were moderately strong and significant.

**Figure 3:**
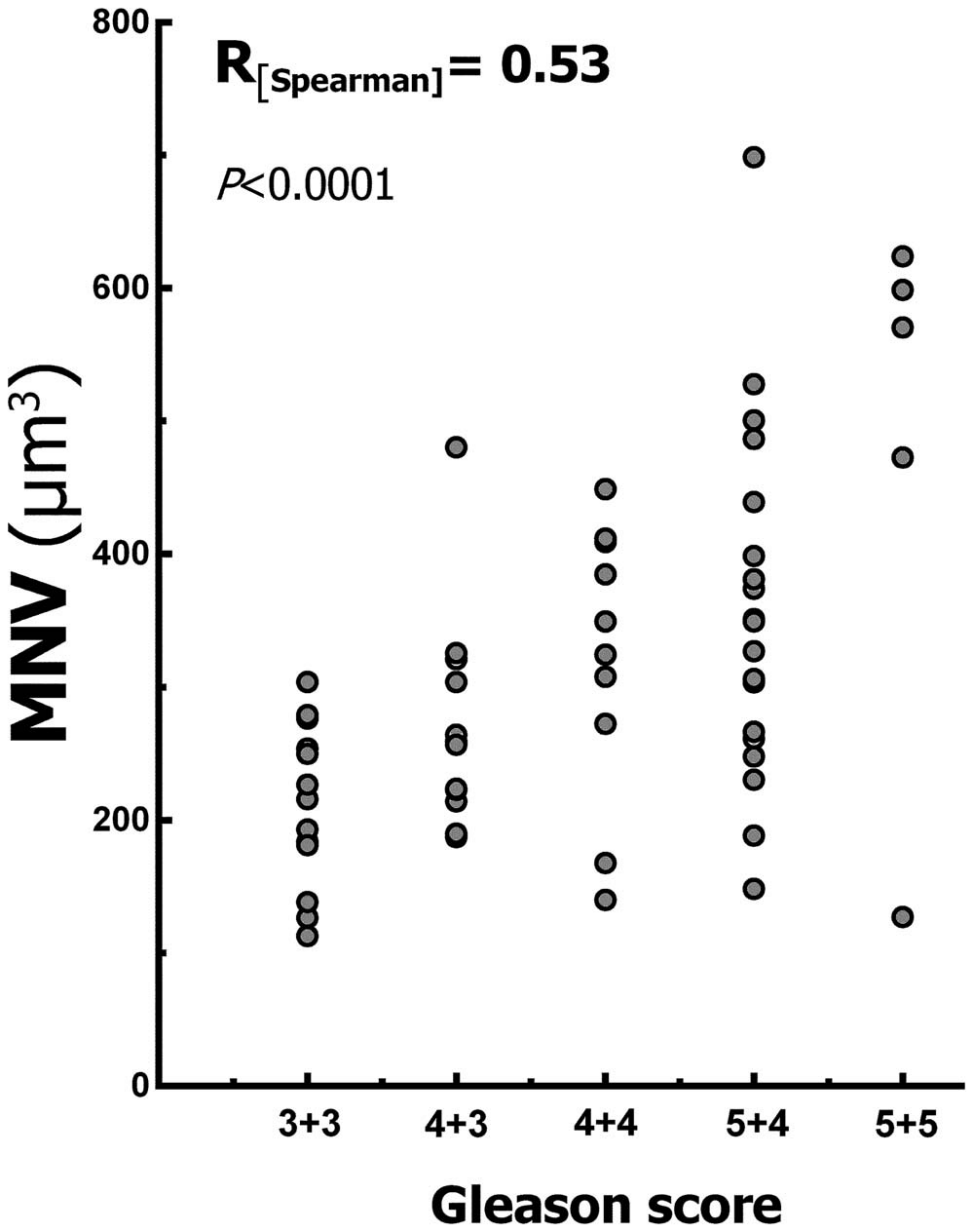
Correlation between Gleason score and mean nuclear volume (MNV). The Spearman coefficient of correlation was moderate and significant for the sample.

- **PSA vs. Gleason scores.** The correlation between PSA and GSc was moderately strong, demonstrating an R_[Spearman]_ value of 0.76 with a 95% CI of 0.62 to 0.85, which was highly significant (*P*<0.0001) (Fig. 2).
- **PSA vs. MNV.** The correlation between PSA and MNV was moderately strong, demonstrating an R_[Spearman]_ value of 0.78 with a 95% CI of 0.65 to 0.86, which was highly significant (*P*<0.0001) (Fig. 2).
- **MNV vs. Gleason primary pattern.** The correlation between MNV and Gpp was moderate, demonstrating an R_[Spearman]_ value of 0.53 with a 95% CI of 0.30 to 0.69, which was highly significant (*P*<0.0001) (Fig. 3).

### Mean nuclear volume

**Figure 4:**
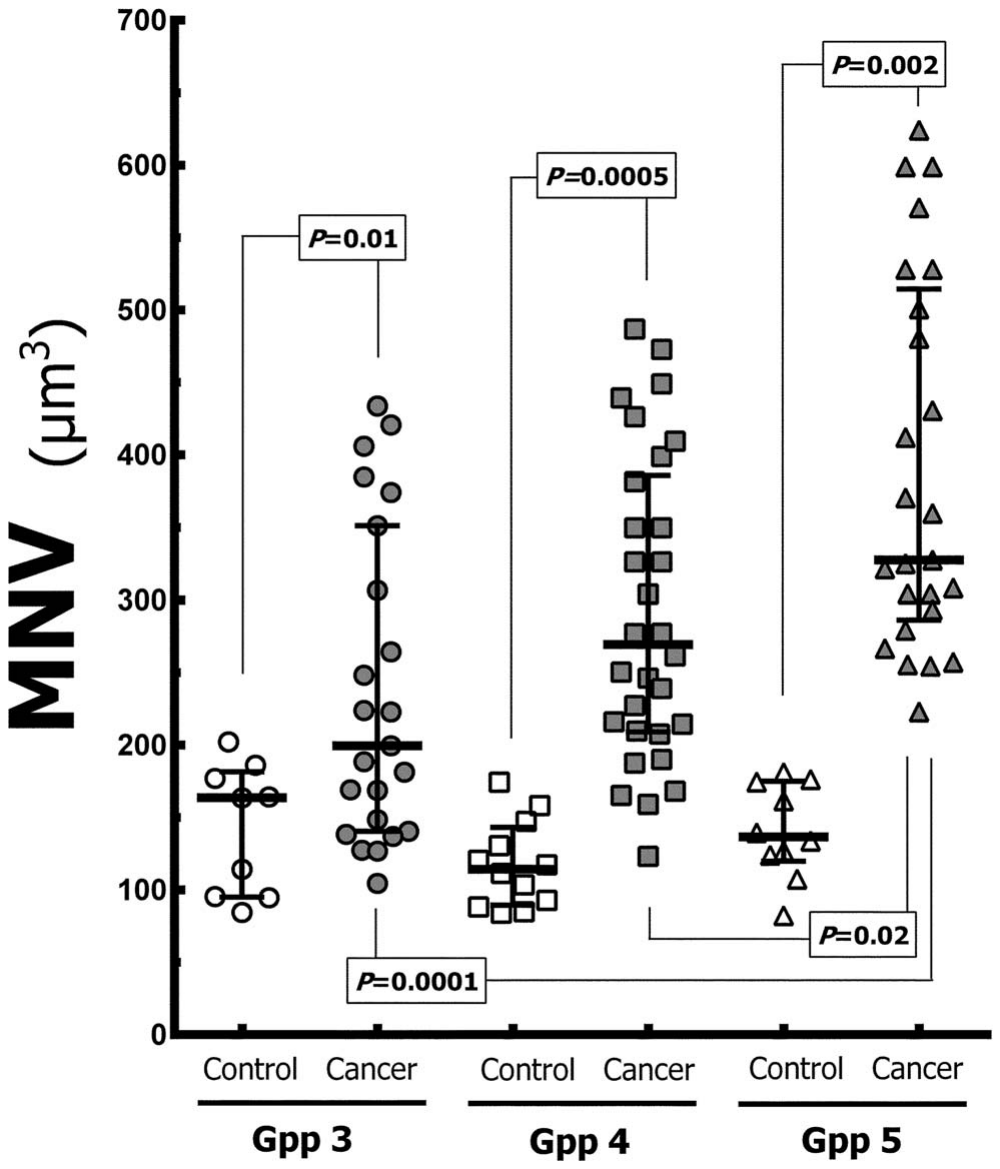
Dispersion of mean nuclear volume (MNV) in the groups with median (horizontal line) and 95% interval confidence noted (see also Table 1). The statistical significance is indicated.

The variability of MNV for the groups is detailed in Fig. 4 and Table 1. The differences were significant when comparing MNV in a Gpp with its control counterparts. MNV was significantly greater in cancerous regions than in paired-control region by 22% in Gpp 3, by 135% in Gpp 4, and by 140% in Gpp 5. There was a continuous increase in MNV in cancerous regions from Gpp 3 to Gpp 5. These differences were significant, and MNV of the G5 group was 64% greater than G3 and 22% greater than G4. Although MNV demonstrated a higher value in G4 compared to G3, this difference was not significant and could be based on sample size.

**Table 1:** The mean nuclear volume (MNV) in the groups.

Score	Control (MNV, µm^3^)		Cancer (MNV, µm^3^)		Variation			
	Median	95% CI	Median	95% CI	%			
3	163.6	94.7 to 186.2	199.6	148.5 to 306.6	C3 *vs.* G3	+22	G3 *vs.* G5	+64
4	114.4	88.3 to 147.4	269.3	216.0 to 349.6	C4 *vs.* G4	+135	G3 *vs.* G4	-
5	136.6	107.2 to 176.2	327.4	304.3 to 480.5	C5 *vs.* G5	+140	G4 *vs.* G5	+22

The significant variations between the Control (C) and the Gleason primary pattern in the cancer groups (G) are indicated.

## Discussion

In the present study, there were significant and moderately strong correlations between PSA and GSc and between PSA and MNV. The correlation between Gpp and MNV was also significant but moderate. These findings indicate that both Gpp and MNV are adjunct parameters in the diagnosis and/or surveillance of PCa. This is extremely relevant because, as mentioned, GSc is a comparative method that is dependent on the training of the pathologist, while MNV is based on counts obtained through design-based stereology.

The Gpp instead the GSc was compared with MNV to obtain the most reliable results possible, as we had selected the most prevalent pattern for each histological sample to avoid biases in studying the surrounding fields when the highest Gleason pattern was used (in several samples these fields were small). With this strategy, there was no difference when comparing the former GSc and the ISUP modifications applied from 2005. However, when PSA was correlated with GSc and MNV, the 2005 ISUP modifications made a difference.

MNV was always significantly different when comparing control regions (non-tumor) with regions containing the tumor, indicating that MNV is an effective alternative to evaluate PCa in cases where immunohistochemistry is unavailable or to increase the reliability of a diagnosis. In addition, MNV value increased from Gpp 3 to Gpp 5 and was different between Gpp 3 and Gpp 5 and between Gpp 4 and Gpp 5. We emphasize that the observation of no difference in MNV between Gpp 3 and Gpp 4 is possibly due to sample size. Further studies could clarify this point in the future.

Conventional and modified Gleason grading both correlated with age, serum PSA and cancer involvement in needle biopsies [15]. Moreover, there was a strong correlation between GSc and tumor volume in well/intermediate differentiated PCa, and given that relatively high amount of PSA per unit volume of cancer are produced, high PSA density was the strongest single predictor of tumor undergrading. However, as higher grade tumors produce less PSA per unit volume, PSA density loses its predictive ability, and other clinical markers of tumor volume such as palpable disease and numbers of positive cores become more predictive [16], [17], as MNV may contribute significantly to the prediction of a biochemical control (PSA) [18].

Studies have shown that the inter-observer reproducibility of the Gleason grading system remains moderate [6], [7], [19]. Although the magnitude of disagreement was rather modest, substantial proportions of biopsy and prostatectomy specimens had different GSc assigned at diagnosis (63% for biopsy and 72% for prostatectomy), compared to those assigned by expert review [20]. We should remark that, in the present study, the material studied came from radical prostatectomies as the first-choice therapy for localized and locally advanced PCa, and GSc was assigned by only one expert pathologist, which certainly tends to minimize the bias in GSc assignment.

The reliable identification of well-differentiated prostatic adenocarcinoma in biopsy specimens remains challenging [21]. To measure this reliability, agreement among pathologists was tested, and the GSc assigned by 15 expert uropathologists for fifteen PCa biopsy samples were compared GSc assigned by 337 members of the European Network of Uropathology. Agreement between expert and member was poor (mean 71.4% in GSc 6, and mean 56.4% in GSc 7 (*P* = 0.009) [22]. The classification of high-grade PCa as GSc 4+3 and GSc 8–10 resulted in higher levels of agreement between biopsy and radical prostatectomy.

In addition, there is abundant literature reporting dissension between the GSc assigned to patients with a needle biopsy diagnosis and after radical prostatectomy [9],[23]. Consequently, there is an overall tendency to undergrade biopsy samples, and this degree of concordance is possibly supported by the number of biopsy cores obtained (ten or more needle biopsy cores increased the proportion of an exact match to 72%) [23] or by the fact that more than one third of patients were found to have been undergraded based on their initial prostate biopsy [9]. On the other hand, the overestimation of GSc in extended prostate biopsies or the presence of a tertiary higher Gleason pattern obtained in a needle biopsy sample, which is found in almost 20% of radical prostatectomies, may subject individuals to unnecessary aggressive treatment [8], [24]. Therefore, the homogeneity of the samples and the report of GSc in the current study should be considered a valid contribution to the classification and diagnosis of PCa.

It should be noted that the present study was performed with samples obtained from radical prostatectomies, and the GSc took into consideration the modifications made by the ISUP in 2005, which attempted to improve the correlation between biopsy and radical prostatectomy. These changes could be the reasons for some differences concerning the correlation between MNV and GSc when comparing findings reported previously (before 2005) [13], when histological samples were taken from needle biopsies, and the present findings.

The study of the pathophysiological changes associated with human diseases involving mutations in nuclear structure has clarified many physiological functions of nuclear structure and organization. Changes in nuclear shapes can be a result of forces acting on the extracellular matrix or of intracellular processes, which are thought to be transmitted to the nucleus via the cellular cytoskeleton [25]. As malignant tumors can alter nuclear shape and increase its size, the estimation of nuclear enlargement in terms of volume in a three-dimensional method appears to be necessary [26].

The updated information from the Brazilian Institute of Geography and Statistics informs that the city of Rio de Janeiro has about 6.32 million people, and then we can accept that 3.16 million are men. The “pyramid of age” of Brazil indicates 9.5% of the population of men has over 50 years of age. Then, in the city of Rio de Janeiro, we can accept that 300,200 men are over 50 years old (which is the age group comparable to the present study of prostate cancer).

Prostate cancer in southeastern Brazil (where is located the city of Rio de Janeiro) has a prevalence of 80.06 cases per 100,000 individuals per year (2014 data from the National Cancer Institute of Brazil). Therefore, we may estimate at 210 the new cases per year of prostate cancer in the city of Rio de Janeiro.

The sample size studied in the present manuscript is a limitation of the survey. However, it is relevant to say that the cases studied account for about 35% of expected new cases in one year in the city of Rio de Janeiro.

Since 1985, pathologists and researchers have explored the possibility of estimating the mean volume of particles of arbitrary shape [14]. A previous study showed an association between MNV and PSA doubling time, which positively correlated MNV with PCa behavior and prognosis [27], suggesting MNV as a predictor of PSA failure for patients with clinically organ-confined disease treated with radical prostatectomy [28]. The findings of the current study are in agreement with this correlation between MNV and PSA levels, as well as MNV and Gpp. In addition, the results of studies combining PSA and estimated tumor volume with estimates of MNV have significantly contributed to the prediction of the pathological stage of PCa, suggesting the use of these three factors to predict the pathological stage of PCa before surgery [29], [30].

## Conclusions

Current data are in favor of the use of MNV associated with GSc and PSA in the assessment of PCa. Estimation of MNV requires a design-based approach with some involvement from mathematics and statistics. Indeed, this can be considered impractical for wide use in the routine evaluation of PCa by the classic pathologist. However, proper study planning, as well as the current availability of equipment and software for morphological quantification, can permit the quick and accurate estimation of MNV as an adjunct parameter for the assessment of PCa.

## Supporting Information

Table S1 **Data organized to calculate the coefficient of correlation of Spearman: Gleason score vs. prostatic specific antigen (PSA, ng/mL), and mean nuclear volume (MNV, µm^3^) vs. PSA.**
(PDF)

Table S2 **Data organized to calculate de coefficient of correlation of Spearman: mean nuclear volume (MNV, µm^3^) in each Gleason primary pattern (G3, G4 or G5) vs. prostatic specific antigen (PSA, ng/mL).**
(PDF)
