# Supplementary material for: Advantages of Evaluating Mean Nuclear Volume as an Adjunct Parameter in Prostate Cancer
Source: PLoS One. 2014 Jul 9;9(7):e102156. doi: 10.1371/journal.pone.0102156 (PMC4090007; doi:10.1371/journal.pone.0102156)
Supplement: Table S1 — Data organized to calculate the coefficient of correlation of Spearman: Gleason score vs. prostatic specific antigen (PSA, ng/mL), and mean nuclear volume (MNV, µm3) vs. PSA. (PDF) [file pone.0102156.s001.pdf]

Data organized to calculate the coefficient of correlation of Spearman

Gleason	PSA	MNV	PSA
6	3.8	184.1	2.0
6	3.9	193.4	2.1
6	3.9	254.4	2.2
6	4.1	255.6	2.4
6	4.1	113.2	2.7
7	4.4	187.5	2.7
7	5.1	259.5	3.0
6	5.5	277.0	3.3
7	5.5	304.3	3.3
7	6.1	190.0	3.4
6	6.3	126.8	3.8
9	6.3	188.7	3.9
6	6.4	138.5	3.9
6	6.5	181.5	4.1
8	6.5	168.1	4.3
6	6.6	216.0	4.4
7	6.6	321.5	4.4
7	6.6	214.4	5.0
6	7.0	250.3	5.0
6	7.0	227.2	5.1
6	7.0	304.3	5.2
8	7.8	140.5	5.5
6	7.9	277.3	5.5
6	8.0	279.3	6.0
9	8.1	148.5	6.3
7	8.5	223.0	6.5
8	8.8	349.6	6.5
7	8.9	224.0	6.6
9	9.3	248.3	6.6
9	10.0	261.6	6.6
7	11.1	326.2	6.6
9	11.1	327.4	6.6
7	11.2	264.5	7.0
9	11.2	374.1	7.0
8	12.0	273.0	7.1
9	12.0	304.1	7.8
9	12.1	306.6	7.9
9	12.4	351.2	8.0
9	12.5	349.6	8.5
8	12.7	308.2	8.5
8	13.3	409.3	8.8
8	13.4	325.0	8.8
9	13.7	381.4	8.9
8	14.0	384.8	11.0
9	14.2	439.3	11.1

Gleason = score
PSA= prostatic specific antigen
MNV= mean nuclear volume

8	14.3
9	14.4
7	14.6
9	14.7
9	14.7
7	15.0
9	15.2
10	17.4
8	18.0
9	18.0
10	18.5
10	19.3
9	21.0
10	22.0
9	23.0
10	26.4

449.0	11.2
528.0	13.7
257.2	13.7
398.8	14.0
266.7	14.0
480.5	14.7
500.9	14.7
472.8	15.0
412.0	16.0
486.9	17.6
624.2	18.0
570.6	21.0
230.7	22.0
598.9	23.0
698.9	26.4
127.5	6.2
