# Supplementary material for: Advantages of Evaluating Mean Nuclear Volume as an Adjunct Parameter in Prostate Cancer
Source: PLoS One. 2014 Jul 9;9(7):e102156. doi: 10.1371/journal.pone.0102156 (PMC4090007; doi:10.1371/journal.pone.0102156)
Supplement: Table S2 — Data organized to calculate de coefficient of correlation of Spearman: mean nuclear volume (MNV, µm3) in each Gleason primary pattern (G3, G4 or G5) vs. prostatic specific antigen (PSA, ng/mL). (PDF) [file pone.0102156.s002.pdf]

DATA organized to calculate the coefficient of correlation of Spearman

MNV G3	PSA	MNV G4	PSA	MNV G5	PSA
264.5	7.0	216.0	4.4	480.5	14.7
306.6	7.9	277.3	5.5	273.0	7.1
225.4	6.6	261.6	6.6	624.2	18.0
188.7	3.9	326.2	12.4	184.1	2.0
148.5	6.3	472.8	15.0	304.3	3.3
351.2	8.0	398.8	14.0	259.5	3.0
138.5	3.9	349.6	6.5	193.4	2.1
384.8	11.0	277.0	3.3	528.0	13.7
374.1	7.0	381.4	8.9	327.4	6.6
269.4	6.4	214.4	5.0	370.4	9.2
168.9	5.1	439.3	11.1	500.9	14.7
223.0	6.5	113.2	2.7	698.9	59.8
224.0	6.6	168.1	4.3	223.5	4.2
127.5	4.3	349.6	8.5	304.3	5.2
181.5	4.1	304.1	7.8	308.2	8.5
140.5	5.5	187.5	2.7	321.5	4.4
126.8	3.8	326.2	6.6	279.3	6.0
248.3	6.6	449.0	11.2	570.6	21.0
169.3	5.3	190.0	3.4	598.9	23.0
104.5	3.7	246.3	6.4	266.7	14.0
137.1	3.1	250.3	5.0	412.0	16.0
199.6	4.9	409.3	8.8	257.2	13.7
		227.2	5.1	230.7	22.0
		209.6	5.6	254.4	2.2
		207.6	6.0	255.6	2.4
		486.8	17.6	325.0	8.8

MNV= mean nuclear volume (μm^3)
G= Gleason primary pattern
